# Pharmacokinetics of Ceftriaxone Encapsulated in Carrier Erythrocytes in Experimental Study

**DOI:** 10.3390/pharmaceutics18091052

**Published:** 2026-08-25

**Authors:** Kulzhan Berikkhanova, Alexandr Gulyayev, Yernur Zakirov, Askhat Zhilkaidarov, Azhar Zhaisanova, Nurgul Daniyeva, Ardak Omarbekov, Gulsara Berikkhanova, Yessenkhan Sultan, Zhannat Zhakiyanova, Gulyash Tanysheva

**Affiliations:** 1 National Laboratory Astana, Nazarbayev University, Astana 010000, Kazakhstan; kberikkhanova@nu.edu.kz (K.B.);; 2 University Medical Center, Nazarbayev University, Astana 010000, Kazakhstan; 3 Professor G.V. Tsoi Scientific and Educational Center of Surgery, Astana Medical University, Astana 010000, Kazakhstan; 4 Department of Clinical Pharmacology and Evidence-Based Medicine, Karaganda Medical University, Karaganda 100017, Kazakhstan; 5 Core Facilities, Nazarbayev University, Astana 010000, Kazakhstan; 6 Department of Higher Mathematics, Faculty of Computer Systems and Professional Education, Saken Seifullin Kazakh Agrotechnical University, Astana 010000, Kazakhstan; 7 Medical Center “Sultan-Medicus”, Almaty 050000, Kazakhstan; 8 Department of Obstetrics and Gynecology Named After A.A. Kozbagarov, Department of Neurology, Ophthalmology and Otorhinolaryngology, Semey State Medical University, Semey 071400, Kazakhstan

**Keywords:** Ceftriaxone, targeted drug delivery, hypoosmotic hemolysis, erythrocyte encapsulation, pharmacocytes, pharmacokinetics, tissue distribution

## Abstract

**Background/Objectives**: Ceftriaxone (Ctx) is a third-generation cephalosporin widely used to treat infections caused by Gram-positive and Gram-negative bacteria. However, its clinical efficacy may be limited by rapid systemic elimination and suboptimal tissue distribution. Erythrocyte-based targeted drug delivery systems (TDDSs) have emerged as a promising approach to prolong drug circulation and enhance site-specific accumulation. This study investigated the pharmacokinetic profile and tissue distribution of ceftriaxone encapsulated in autologous erythrocytes (RBC-Ctx) compared with free ceftriaxone (Free-Ctx) following intravenous administration in rats. **Methods**: Ceftriaxone was encapsulated into autologous rat erythrocytes using a hypoosmotic hemolysis loading technique. Drug-loaded erythrocytes are called pharmacocytes. Adult male Wistar rats received a single intravenous injection of Free-Ctx or RBC-Ctx at an equivalent ceftriaxone dose of 340 mg/kg. Plasma samples were collected over 24 h for pharmacokinetic analysis, while the liver, spleen, lungs, kidneys, heart, pancreas, and skeletal muscle were harvested at 1 and 12 h for tissue distribution studies. Ceftriaxone concentrations were quantified by high-performance liquid chromatography with UV detection. **Results**: Erythrocyte encapsulation significantly modified the pharmacokinetic behavior of ceftriaxone. Compared with Free-Ctx, RBC-Ctx prolonged the elimination half-life (4.4 ± 0.6 vs. 1.8 ± 0.1 h), increased systemic exposure (AUC_0_–last, 1.6 ± 0.1 vs. 1.2 ± 0.2 mg·h/mL), reduced total body clearance (218.2 ± 11.0 vs. 294.0 ± 48.8 mL/h/kg), and increased the apparent volume of distribution at steady state (688.3 ± 61.0 vs. 435.0 ± 23.1 mL/kg). In addition, RBC-Ctx was associated with a distinct relative tissue-distribution pattern of ceftriaxone, particularly in reticuloendothelial system-rich organs such as the liver and spleen, while ceftriaxone remained detectable in several tissues at 12 h after administration. In contrast, ceftriaxone concentrations following Free-Ctx declined markedly or became undetectable over the same period. **Conclusions**: Encapsulation of ceftriaxone into autologous erythrocytes substantially prolonged systemic circulation, enhanced drug exposure, reduced clearance, and altered the relative tissue distribution of ceftriaxone. These findings demonstrate that erythrocyte-based carriers effectively modulate ceftriaxone pharmacokinetics and tissue distribution, supporting their potential as a targeted antibiotic delivery platform for improving antimicrobial therapy, particularly for infections involving reticuloendothelial system-associated tissues. Further studies in experimental models of infection and inflammation are warranted to evaluate therapeutic efficacy under pathological conditions and to optimize this delivery strategy.

## 1. Introduction

Targeted drug delivery remains one of the urgent challenges in pharmacology. The use of carriers to transport drugs within the body promises more selective distribution and higher accumulation of drugs at specific target sites. This expectation drives the ongoing progress and innovation in the development of targeted drug delivery systems (TDDSs) [1,2,3]. This challenge is particularly relevant in the fields of chemotherapy and antimicrobial therapy, where targeted drug delivery is pursued not only to enhance therapeutic efficacy but also to overcome drug resistance through the selective accumulation of therapeutics at the site of action. Owing to their unique physicochemical properties, TDDSs offer innovative strategies to circumvent conventional bacterial defense mechanisms, including membrane permeability barriers, efflux pump-mediated drug extrusion, and biofilm formation [4,5,6]. Nanomaterials were initially proposed as promising platforms for TDDSs because of their ability to enhance drug solubility and stability, minimize off-target toxicity, and facilitate targeted delivery. Nevertheless, despite their broad therapeutic potential, nanoparticles possess inherent limitations. In particular, their high surface reactivity may contribute to undesirable side effects. Moreover, multiple factors, including particle size, shape, surface charge, chemical composition, physicochemical characteristics, stability, administered dose, and host immune competence, influence nanoparticle–cell interactions and ultimately determine their biological fate [7]. In addition, nanoparticles are often rapidly recognized and cleared by the immune system, thereby reducing drug bioavailability at the target site and limiting therapeutic effectiveness [8,9]. In this context, autologous blood cells have emerged as particularly attractive candidates for TDDSs. Blood cell–based drug delivery systems offer several advantages over conventional polymer-based platforms. These systems typically employ erythrocytes (red blood cells), leukocytes, and platelets as natural carriers. Both intact blood cells and their derived membranes can be utilized for the delivery of antibacterial and other therapeutic agents. Importantly, blood cells possess inherent biological properties that allow them to evade or navigate many of the physiological and pathological barriers that restrict nanoparticle performance in vivo, thereby enabling more efficient and targeted delivery of therapeutics to diseased tissues [10,11].

Among autologous blood cell-based TDDSs, erythrocytes exhibit particularly advantageous characteristics. Extensive research over the years has explored erythrocytes as drug delivery vehicles, primarily because they are the most abundant cell type in the bloodstream and possess remarkable properties, including a long lifespan of 100–120 days, low immunogenicity, excellent biocompatibility, and high deformability [12,13,14,15]. Frequently referred to as “intelligent delivery systems” [16], erythrocytes can function as biological or hybrid drug carriers owing to their intrinsic advantages and unique physiological characteristics. This innovative strategy has the potential to enhance drug biocompatibility and pharmacokinetic performance while enabling targeted delivery.

Antibiotics encapsulated within erythrocytes have been proposed as a promising strategy for the treatment of intracellular pathogens and may, under specific conditions, contribute to overcoming antibiotic resistance. Potential advantages over conventional administration of free antibiotics include reduced systemic dosing requirements, decreased toxicity, prolonged and targeted delivery of antibiotics at elevated concentrations to sites of pathogen replication, and improved therapeutic outcomes [17].

Despite their apparent promise, erythrocyte-based targeted drug delivery systems (TDDSs) remain at a relatively early stage of development. Nevertheless, erythrocyte-mediated drug delivery has emerged as a rapidly growing area of research with considerable translational potential.

Encapsulation of antibiotics within erythrocyte ghosts is expected to fundamentally alter key pharmacokinetic properties of the drug, although experimental evidence supporting this hypothesis remains limited. Indeed, both in vitro and in vivo studies investigating antibiotic delivery using erythrocyte-based or other cell-derived carriers are still relatively scarce. To date, only a limited number of studies have reported the use of erythrocytes as carriers for antibiotics, including gentamicin-loaded erythrocytes [18] and amikacin-loaded erythrocytes [17,19]. The authors [20] examined recent advances in the clinical application of red blood cell-based (RBC-based) targeted drug delivery systems. In study [21], Interleukin-1β (IL-1β) encapsulated in red blood cell (RBC) ghosts showed improved pharmacokinetics after a single intravenous injection, with prolonged half-life, reduced clearance, and increased accumulation in the liver, spleen, and lungs compared to free IL-1β.

To the best of our knowledge, the pharmacokinetics of ceftriaxone encapsulated within erythrocyte ghosts has not yet been characterized, despite the widespread clinical use and therapeutic importance of this antibiotic.

To address this knowledge gap, the present study sought to identify the key pharmacokinetic parameters governing the in vivo behavior of ceftriaxone incorporated into an erythrocyte-based cellular delivery system in experimental animals.

Thus, this study investigated the pharmacokinetic profile and tissue distribution of ceftriaxone encapsulated in autologous erythrocytes (RBC-Ctx) compared with free ceftriaxone (Free-Ctx) following intravenous administration in rats.

Encapsulation of ceftriaxone into autologous erythrocytes substantially prolonged systemic circulation, enhanced drug exposure, reduced clearance, and altered the relative tissue distribution of ceftriaxone. These findings demonstrate that erythrocyte-based carriers effectively modulate ceftriaxone pharmacokinetics and tissue distribution, supporting their potential as a targeted antibiotic delivery platform for improving antimicrobial therapy, particularly for infections involving reticuloendothelial system-associated tissues.

## 2. Materials and Methods

### 2.1. Experimental Animals

The experiments were conducted on adult male Wistar rats weighing 250 ± 20 g. We selected a group of animals consisting of only six individuals per group as an empirical compromise between statistical accuracy and minimizing animal costs. The sample size directly reflects the fundamental principle of bioethics: Reduction. This sample size is traditionally used in pharmacokinetic studies and allows us to obtain statistically reliable values with the smallest possible sample size. The animals were housed in individually ventilated polycarbonate cages under standard laboratory conditions, including a controlled temperature of 22 ± 2 °C, relative humidity of 55 ± 10%, and a 12:12 h light/dark cycle. The rats were fed ad libitum with unrestricted access to standard food and water. Prior to the experiments, the animals were acclimatized for at least one week. All procedures were carried out with appropriate anesthesia, under isoflurane inhalation anesthesia using a RWD R540 veterinary anesthesia system for rodents (RWD Life Science, Shenzhen, China) [22]. All procedures were performed in accordance with the guidelines for the care and use of laboratory animals. All experiments involving animals were approved by the Institutional Animal Care and Use Committee (IACUC) at Nazarbayev University (approval ID: 4/13112024).

### 2.2. Isolation of Erythrocytes (RBCs)

Fresh whole blood was collected from rats via tail vein puncture and maintained at 4 °C for a short period prior to processing, as previously described [23]. Erythrocytes were isolated by centrifugation of whole blood at 1000 rpm for 5 min using a Megafuge 16R centrifuge (Thermo Scientific, Dreieich, Germany). Following centrifugation, the plasma and buffy coat, containing leukocytes and platelets, were carefully removed and discarded, leaving the erythrocyte-rich pellet. The erythrocyte-enriched pellet was then washed three times with physiological saline to remove residual plasma proteins and other non-erythrocyte components following the general erythrocyte isolation procedure described by Brenner et al. [24]. The purified erythrocytes were subsequently used for the preparation of ceftriaxone-loaded erythrocytes (RBC-Ctx).

### 2.3. Preparation of RBC-Ctx Pharmacocytes (Ceftriaxone-Loaded Erythrocytes)

Ceftriaxone was loaded into isolated erythrocytes using the osmotic swelling method, which induces the formation of transient pores in the erythrocyte membrane. Washed erythrocytes are loaded with therapeutic agents through these temporary membrane pores generated during osmotic swelling under hypotonic conditions in the presence of a high drug concentration [25,26]. The osmotic swelling procedure was performed with a modification of a previously described method [27]. Briefly, 0.5 mL of the erythrocyte-rich fraction obtained from 1.0 mL of whole blood was mixed with a fivefold volume (2.5 mL) of ultrapure distilled water (UltraPure, Invitrogen, Paisley, UK) pre-cooled to 0 °C and gently mixed. The suspension was then centrifuged at 7000 rpm for 20 min at 4 °C (Eppendorf 5420, Hamburg, Germany). The resulting erythrocyte ghosts were incubated with a ceftriaxone solution prepared by dissolving 85 mg of ceftriaxone in 1 mL of distilled water for 15–20 min at 4 °C. To restore isotonic conditions and facilitate membrane resealing, 1/9 volume of sterile 10% NaCl solution pre-cooled to 0 °C was added to the suspension. The mixture was gently mixed and incubated at 37 °C for 20 min in a temperature-controlled incubator (BINDER, Tuttlingen, Germany). The resulting suspension of ceftriaxone-loaded erythrocytes (RBC-Ctx) was subsequently used for intravenous administration to experimental animals.

### 2.4. Assessment of Erythrocyte Membrane Integrity

To differentiate between loaded and unloaded erythrocytes and to evaluate the effect of the loading procedure on erythrocyte membrane integrity, the erythrocyte sedimentation rate (ESR) was determined according to the Westergren method [28]. The Westergren tube was securely mounted in a vertical position on a Westergren stand, ensuring the absence of leakage throughout the measurement period. The tube was then left undisturbed for 1 h, after which the sedimentation results were recorded. For subsequent analysis, the distance traveled by the sedimenting erythrocytes and the height of the clear cell-free supernatant column at the top of the tube were measured and expressed in millimeters.

### 2.5. Microscopic Morphological Examination of Erythrocytes

The morphology of ceftriaxone-loaded erythrocytes and unloaded (empty) erythrocytes was evaluated using light microscopy. Erythrocyte samples were washed twice with distilled water, and 10–15 μL of the suspension was spread onto a glass slide to prepare a uniform blood smear. The smear was air-dried, fixed with 99.8% methanol, and allowed to air-dry again. The slides were then stained with a commercially available Giemsa stain diluted 1:9 with distilled water (Giemsa: distilled water). Staining was performed for 15 min, after which the slides were rinsed with water and air-dried. The prepared slides were examined under a light microscope to assess erythrocyte morphology before and after the different treatment procedures under a Carl Zeiss Axioscope 5 light microscope (Carl Zeiss AG, Oberkochen, Germany).

### 2.6. Pharmacokinetic and Tissue Distribution Studies

The experiments were conducted on adult male Wistar rats weighing 250 ± 20 g. Animals were randomly assigned to two experimental groups: Free-Ctx and RBC-Ctx. For the pharmacokinetic study, six animals were included in each treatment group (*n* = 6 per group). For the tissue distribution study, separate cohorts of six animals per treatment group were used at each tissue collection time point (*n* = 6 per group at 1 h and *n* = 6 per group at 12 h). Thus, a total of 36 animals were used in the pharmacokinetic and tissue distribution experiments. The sample size of six animals per group for each experimental cohort was selected in accordance with the Reduction principle of the 3Rs as an empirical compromise between obtaining adequate statistical precision and minimizing animal use. This sample size is commonly used in exploratory pharmacokinetic studies and was considered sufficient for obtaining reliable pharmacokinetic and tissue distribution estimates while limiting animal use. Rats in the Free-Ctx group received a single intravenous injection of free ceftriaxone dissolved in physiological saline via the tail vein at a dose of 340 mg/kg. Rats in the RBC-Ctx group received a single intravenous injection of ceftriaxone-loaded carrier erythrocytes suspended in physiological saline via the tail vein at an equivalent ceftriaxone dose of 340 mg/kg. To evaluate the pharmacokinetic profile of ceftriaxone, approximately 500 μL of blood was collected from anesthetized rats into EDTA-containing tubes at 10 min, 1 h, 3 h, 6 h, 9 h, and 24 h following administration. At the end of the study (24 h post-administration), animals were euthanized by carbon dioxide overdose (AE0904-1; Open Science Research and Production Company LLC, Moscow, Russia). Blood samples were immediately centrifuged at 4000 rpm for 15 min at 4 °C using an Eppendorf Centrifuge 5430 (Eppendorf, Hamburg, Germany). The plasma fraction was carefully separated and stored at −80 °C until analysis [29,30]. For tissue distribution studies, subsets of animals from both the Free-Ctx and RBC-Ctx groups were euthanized at 1 h and 12 h after administration of the test formulations. Samples of the kidneys, liver, spleen, lungs, heart, pancreas, and skeletal muscle were collected. The organs were rinsed with ice-cold physiological saline to remove residual blood, weighed, and immediately stored at −80 °C until further analysis.

### 2.7. Quantification of Ceftriaxone in Biological Samples by High-Performance Liquid Chromatography-Ultraviolet (HPLC-UV)

#### 2.7.1. HPLC–UV Analysis

Quantitative determination of ceftriaxone in biological samples was performed using a Dionex Ultimate 3000 UHPLC system equipped with a UV detector (Thermo Fisher Scientific, Waltham, MA, USA). Data acquisition and processing were carried out using Chromeleon Chromatography Data System software version 7.2.10 (Thermo Fisher Scientific). Chromatographic separation was achieved on a Hypersil GOLD C18 analytical column (150 mm × 2.1 mm, 1.9 μm particle size; Thermo Fisher Scientific). The mobile phase consisted of methanol and 10 mM phosphoric acid (25:75, *v*/*v*), delivered isocratically at a flow rate of 0.15 mL/min. Prior to use, the mobile phase was filtered through a 0.45 μm membrane filter. Analyses were performed at 25 °C with UV detection at 260 nm. The injection volume was 2 μL, and the total run time was 12 min.

The chromatographic conditions were optimized based on the spectral characteristics of the mobile-phase components. The mobile phase consisted of 10 mM aqueous phosphoric acid solution and methanol at a ratio of 75:25 (*v*/*v*). Phosphoric acid was selected as the aqueous phase due to its minimal UV absorbance, thereby reducing baseline interference during detection. Among the tested conditions, a methanol/10 mM phosphoric acid mixture (25:75, *v*/*v*) provided the most efficient separation and optimal peak characteristics for ceftriaxone analysis in plasma samples and was therefore selected for subsequent analyses. Under the optimized chromatographic conditions, ceftriaxone exhibited a retention time of approximately 8.07 min.

#### 2.7.2. Method Validation

The linearity of the HPLC–UV method was evaluated using ceftriaxone sodium standard solutions prepared over the concentration range of 0.5–50 mg/L. Briefly, a 1000 mg/L (1000 ppm) stock solution of ceftriaxone sodium was prepared by accurately weighing the ceftriaxone sodium reference standard and dissolving it in physiological saline. The stock solution was subsequently diluted with physiological saline to obtain a 100 mg/L (100 ppm) working solution. A series of ceftriaxone calibration standards covering the concentration range of 0.5–50 mg/L were then prepared by appropriate dilution of the 100 mg/L working solution. The calibration standards were prepared directly in physiological saline and analyzed by HPLC with UV detection under the same analytical conditions as the study samples. Therefore, the calibration curve used in this study represents a solution-based calibration rather than a matrix-matched calibration. Calibration curves were constructed by plotting peak area against analyte concentration. Excellent linearity was observed throughout the investigated range, with a correlation coefficient (r) of 0.9999, indicating high analytical accuracy and reproducibility. The analytical performance characteristics of the method, including the linear dynamic range, regression parameters, limit of detection (LOD), limit of quantification (LOQ), and relative standard deviation (RSD), are summarized in Table 1.

The validated method demonstrated excellent linearity, sensitivity, precision, and reproducibility, confirming its suitability for the quantitative determination of ceftriaxone in biological matrices, including rat plasma and tissue samples.

### 2.8. Sample Preparation for HPLC Analysis

Plasma samples were prepared using a protein precipitation method with acetonitrile (LC–MS grade, Fluka, Germany), adapted from the procedure described by Lin et al. [30]. Briefly, 400 μL of chilled acetonitrile was added to 200 μL of plasma (2:1, *v*/*v*). The mixture was vortex-mixed thoroughly and centrifuged at 9300× *g* for 10 min at 4 °C. The resulting supernatant was collected and subjected to HPLC analysis. Tissue samples (kidneys, liver, spleen, lungs, heart, pancreas, and skeletal muscle) were homogenized using a Potter homogenizer in ice-cold phosphate-buffered saline (PBS, pH 7.2) at a tissue-to-buffer ratio of 1:10 (*w*/*v*; 200 mg tissue in 2 mL PBS). An aliquot of 1 mL of the homogenate was mixed with an equal volume of acetonitrile, vortexed, and centrifuged under the same conditions (9300× *g*, 10 min, 4 °C) to precipitate proteins and particulate matter. The obtained supernatants were filtered through 0.22 μm syringe filters (Isolab, Eschau, Germany) and transferred into HPLC vials (9 mm, PTFE/silicone septa; Supelco, Bellefonte, PA, USA) for subsequent chromatographic analysis. This sample preparation procedure ensured efficient protein removal and minimized matrix interference during quantitative determination of ceftriaxone in plasma and tissue homogenates.

### 2.9. Pharmacokinetic Analysis

Plasma concentration–time data obtained from the Free-Ctx and RBC-Ctx groups were analyzed using non-compartmental analysis (NCA) in PKanalix software (v2024R1, Lixoft^®^, Antony, France) [31]. The maximum plasma concentration (Cmax) and the time to reach maximum concentration (Tmax) were determined directly from the observed data. The terminal elimination rate constant (kel) was estimated by linear regression of the terminal log-linear portion of the concentration–time curve using the last 3–4 measurable concentration points. The elimination half-life (T1/2) was calculated as ln(2)/kel. The area under the plasma concentration–time curve from time zero to the last measurable concentration (AUC_0_–t) was calculated using the linear trapezoidal method. The residual area from the last measurable concentration to infinity (AUCt–∞) was estimated as Clast/kel, where Clast represents the final quantifiable plasma concentration. Total systemic exposure (AUC0–∞) was calculated as the sum of AUC0–t and AUCt–∞. The mean residence time (MRT) was determined as the ratio of the area under the first-moment curve (AUMC) to AUC0–∞.

To further characterize ceftriaxone disposition, compartmental pharmacokinetic modeling was performed separately using Monolix software (v2024R1, Lixoft^®^, France) based on a population approach. The Free-Ctx concentration–time profile was described using a one-compartment model with intravenous bolus administration, whereas the RBC-Ctx profile was described using a two-compartment model. For the RBC-Ctx model, the structural parameters included clearance (Cl), central volume of distribution (V1), intercompartmental clearance (Q), and peripheral volume of distribution (V2). The intercompartmental transfer rate constants were derived as k12 = Q/V1 and k21 = Q/V2. Standard errors and relative standard errors (%RSE) of the population parameter estimates were obtained from the Fisher information matrix. The precision of the derived k12 and k21 estimates was evaluated using the delta method. Model performance was assessed using observed-versus-predicted plots, individual weighted residual diagnostic plots, and a visual predictive check (VPC). Non-compartmental and compartmental model results are reported separately to avoid mixing parameters derived using different analytical approaches.

The distribution of ceftriaxone in organs was assessed at 1 and 12 h after administration of the study drug. Concentrations were measured in the liver, kidneys, spleen, heart, lungs, pancreas, and skeletal muscle. To evaluate relative tissue distribution, the tissue-to-plasma ratio (T/P ratio) was calculated at 1 h after administration, which was the time point at which both tissue and plasma measurements were available. The tissue-to-plasma distribution coefficients (T/P ratios) were calculated at 1 h using the following formula:

(1) $$concentration\;gradient=\frac{tissue\;concentration}{plasma\;concentration}$$

where tissue concentration is the mean analytical value obtained for the corresponding tissue and plasma concentration is the mean plasma ceftriaxone concentration at 1 h.

The encapsulation efficiency of ceftriaxone in erythrocytes was determined using a previously described method [32]. Briefly, 1 mL of ceftriaxone-loaded erythrocytes was centrifuged, and the pellet containing the loaded erythrocytes was collected for drug quantification. The pellet was resuspended in 1 mL of distilled water and incubated at 40 °C for 20 min to induce hemolysis. The resulting suspension was then filtered through a 0.22 μm membrane filter. The concentration of ceftriaxone in the filtrate was quantified by high-performance liquid chromatography (HPLC). The percentage of ceftriaxone encapsulation was calculated using the following equation:

(2) $$\%age\;content\;of\;drug=\frac{Area\;of\;sample\times weight\;of\;standart\times 5}{Area\;of\;standart\times 25\times weight\;of\;sample}\times 100$$

### 2.10. Drug Release Profile

The release of ceftriaxone from erythrocytes was evaluated using the membrane dialysis method as previously described [33]. Briefly, 1 mL of the ceftriaxone-loaded erythrocyte suspension was measured, diluted with 5 mL of phosphate-buffered saline (PBS, pH 7.4), and then transferred into a dialysis membrane. The dialysis bag was immersed in 15 mL of PBS. The system was incubated at 37 °C with continuous mixing at 50 rpm. Samples were collected at predetermined time intervals and replaced with an equal volume of fresh release medium. The collected samples were analyzed by high-performance liquid chromatography (HPLC) to determine the release profile of ceftriaxone from the erythrocytes.

### 2.11. Statistical Analysis

GraphPad Prism 10 (Version 10.4.2, GraphPad Software, Boston, MA, USA) was used for statistical analyses [34]. Normality was assessed using the Shapiro–Wilk test, and homogeneity of variances was evaluated using the Brown–Forsythe test. For comparisons between two independent groups, an unpaired two-tailed Student’s *t*-test was used for normally distributed data with equal variances [35,36]. Welch’s *t*-test was used for normally distributed data with unequal variances, whereas the Mann–Whitney U test was used when the normality assumption was not met. Differences were considered statistically significant at *p* < 0.05. Data are presented as mean ± SD unless otherwise specified.

## 3. Results

### 3.1. HPLC Method Validation

**Figure 1:**
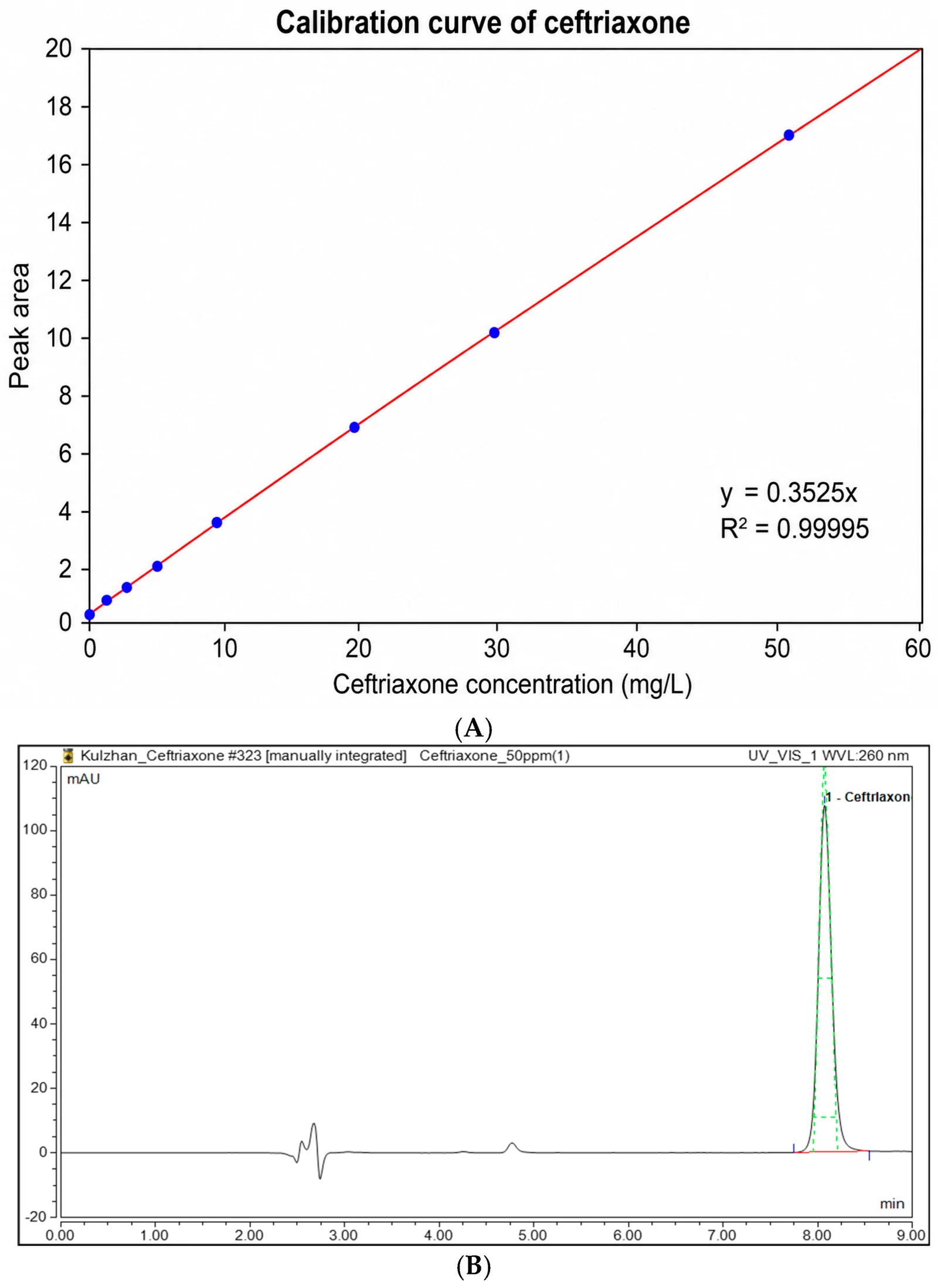
Calibration curve (**A**) and representative chromatogram (**B**) for ceftriaxone determination.

The HPLC method was successfully validated for the quantitative determination of ceftriaxone in erythrocytes in accordance with ICH guidelines with respect to key analytical parameters, namely accuracy, linearity, range, precision, and robustness. The results demonstrated that all system suitability parameters were within the specified limits, confirming the reliability of the developed method. Linearity was established over the concentration range of 0.5–50 mg/L, and a calibration curve was constructed with a correlation coefficient (R^2^) of 0.99995. This indicated that the analytical procedure followed Beer’s law. Specificity assessment demonstrated that ceftriaxone was selectively detected without interference from placebo components, as only the standard solution produced a ceftriaxone peak at a retention time of 8.073 min, as shown in Figure 1, whereas no peak was observed in the placebo sample. The method also demonstrated good reproducibility when repeated at different times of the day (between 10:00 a.m. and 5:00 p.m.) and when repeated on different days, confirming the accuracy of the analysis. The method also exhibited good repeatability, with acceptable intra-day and inter-day variability, confirming the precision of the assay. Figure 1 presents the HPLC calibration curve demonstrating linearity over the range of 0.5–50 mg/L, as well as a representative HPLC chromatogram showing the ceftriaxone retention peak at 8.073 min.

### 3.2. Isolation of Erythrocytes and Preparation of RBC-Ctx

**Figure 2:**
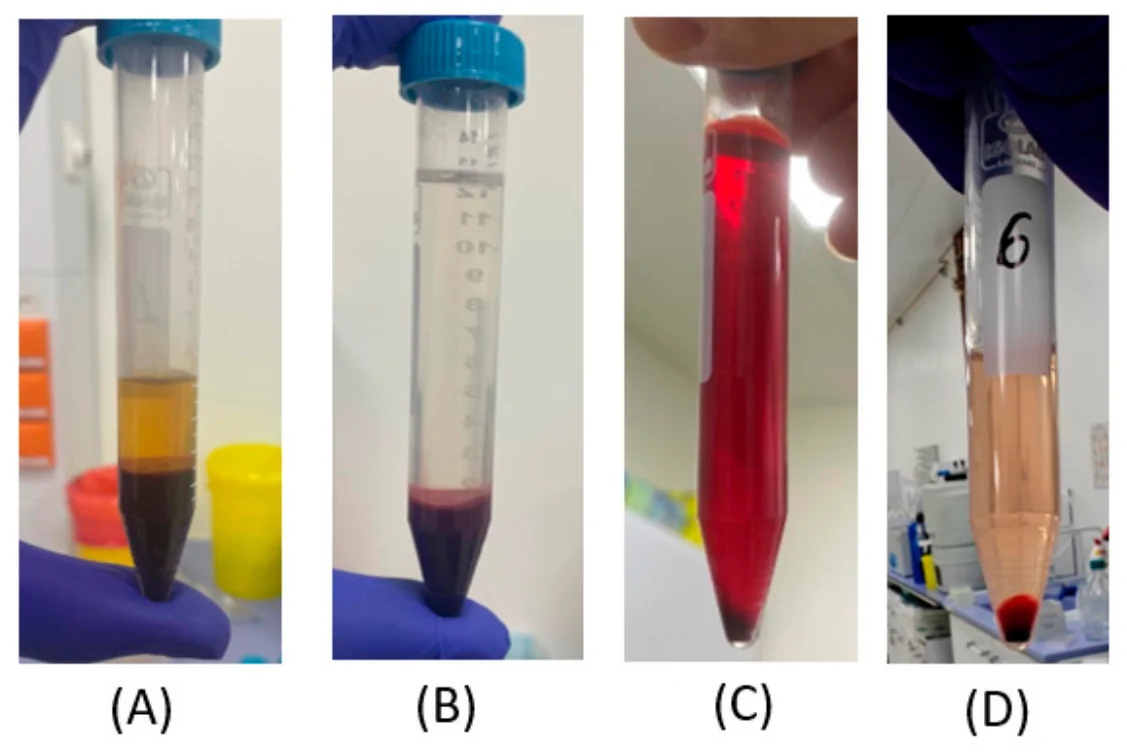
Main stages of ceftriaxone encapsulation into erythrocytes. (**A**) Separation of blood components; (**B**) isolated erythrocytes; (**C**) hemoglobin removal from erythrocytes by hypoosmotic hemolysis; (**D**) ceftriaxone-loaded erythrocytes (RBC-Ctx).

Erythrocytes were isolated from the blood of experimental animals by centrifugation. Ceftriaxone was subsequently loaded into freshly isolated erythrocytes. Figure 2 illustrates the separation of blood components, erythrocyte isolation, and the preparation of RBC-Ctx. The resulting RBC-Ctx were subsequently characterized using multiple parameters. The loading efficiency of ceftriaxone, quantified by HPLC, showed a mean value of 61.9%.

**Figure 3:**
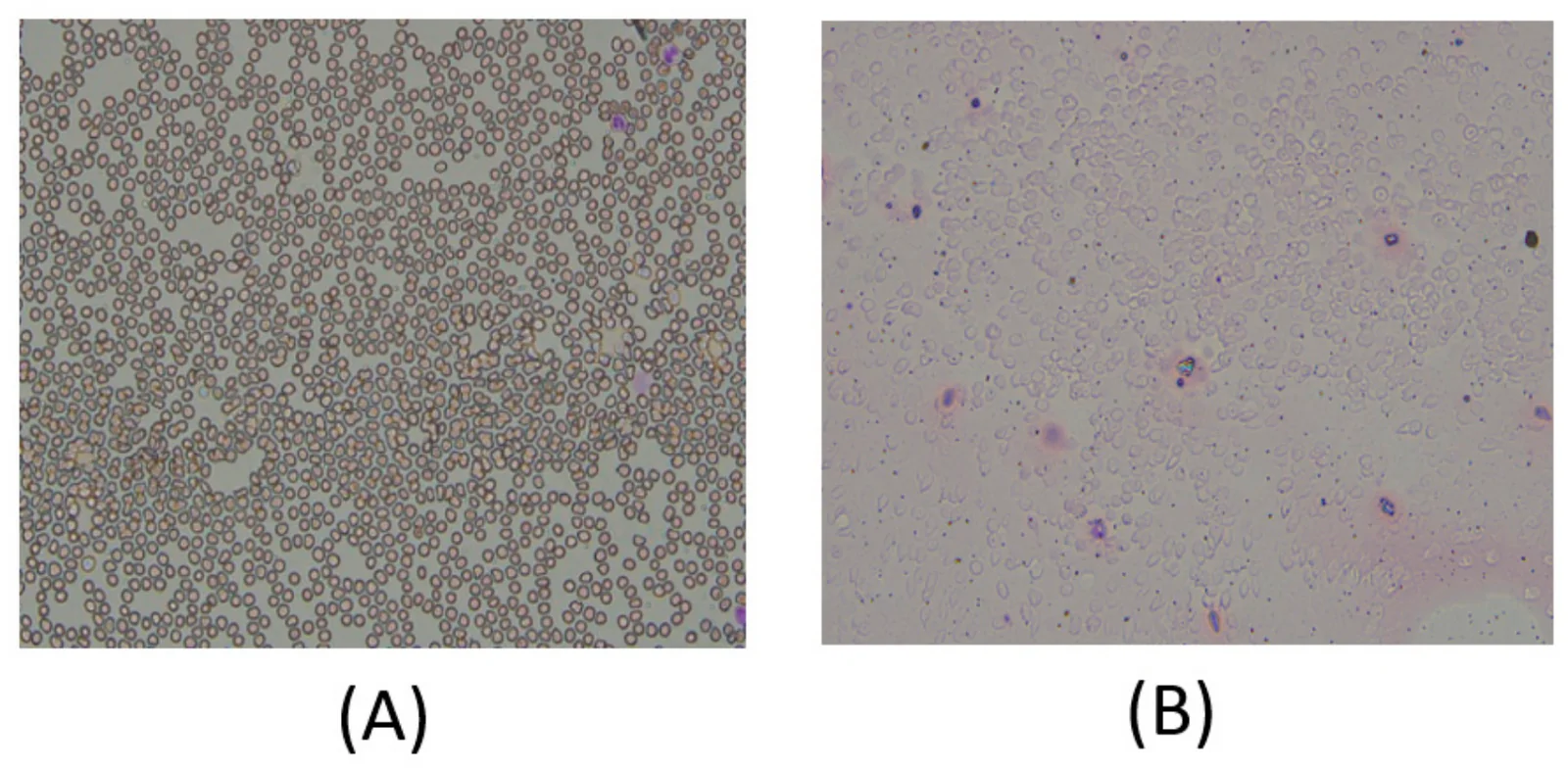
Representative optical microscopy images demonstrating morphological differences before and after drug loading. Stained normal rat erythrocytes (**A**). Ceftriaxone-loaded rat erythrocytes (**B**). Images were obtained using a Carl Zeiss Axioscope 5 light microscope. Original magnification ×40. Romanowsky–Giemsa staining was performed.

Representative optical microscopy images obtained before and after drug loading are shown in Figure 3.

Both ceftriaxone-loaded and normal erythrocytes were examined to assess morphological differences. Ceftriaxone-loaded erythrocytes appeared noticeably enlarged compared with normal cells. The central pallor characteristic of normal erythrocytes was absent in the drug-loaded cells, suggesting successful encapsulation of ceftriaxone within the intracellular compartment. Furthermore, following hypotonic treatment, the erythrocyte membrane appeared slightly roughened and exhibited mild morphological alterations compared with the smooth and regular morphology observed in control erythrocytes.

### 3.3. Erythrocyte Sedimentation Rate

The erythrocyte sedimentation rate (ESR) differed between unloaded and ceftriaxone-loaded erythrocytes. While unloaded erythrocytes exhibited ESR values within the normal range, ceftriaxone-loaded erythrocytes showed a substantially higher ESR of 63 mm/h. This value exceeded the normal erythrocyte ESR range (0–15 mm/h), indicating that the loading procedure altered the sedimentation properties of the erythrocytes, consistent with the morphological changes observed microscopically after ceftriaxone encapsulation.

### 3.4. Release Profile of Ceftriaxone from RBC-Ctx

**Figure 4:**
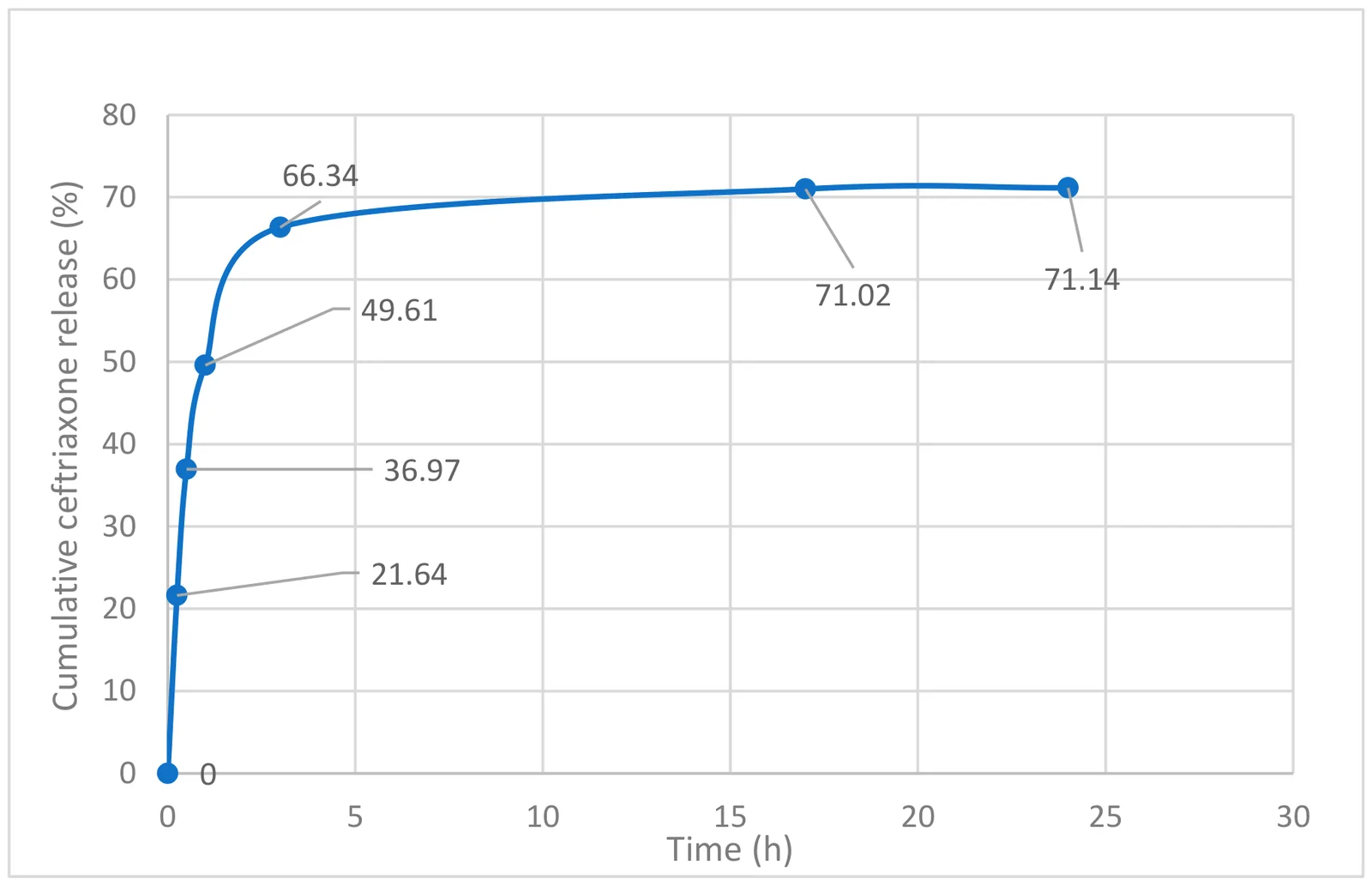
Cumulative in vitro release of ceftriaxone from ceftriaxone-loaded erythrocytes (RBC-Ctx) during 24 h of dialysis. Cumulative release is expressed as the percentage of the initial ceftriaxone amount.

The in vitro release profile of ceftriaxone from RBC-Ctx was assessed over 24 h using phosphate-buffered saline (PBS, pH 7.4) as the release medium. Drug release was evaluated by the membrane dialysis method, and the resulting release profile is shown in Figure 4.

As shown in Figure 4, the cumulative release of ceftriaxone from RBC-Ctx increased rapidly during the first 3 h, reaching approximately 66.3% of the initial drug amount. Thereafter, the release rate markedly decreased and the cumulative release approached a plateau, reaching approximately 71.1% at 24 h. Based on the initial ceftriaxone amount of 128.205 mg contained in 1 mL of pharmacocytes and the dialysis medium volume of 15 mL, the total amount of ceftriaxone released over 24 h was approximately 91.2 mg. Several mechanisms have been proposed to explain drug release from encapsulated erythrocytes. We agree with the hypothesis that post-hypotonic treatment may reduce membrane integrity due to pore formation [37]. We suggest that the mechanism of drug release from erythrocyte-encapsulated formulations may differ between the above-described in vitro conditions and in vivo conditions, where additional uptake of RBC-Ctx pharmacocytes by the reticuloendothelial system (RES) contributes to the process.

### 3.5. Pharmacokinetic Parameters of Ceftriaxone

**Figure 5:**
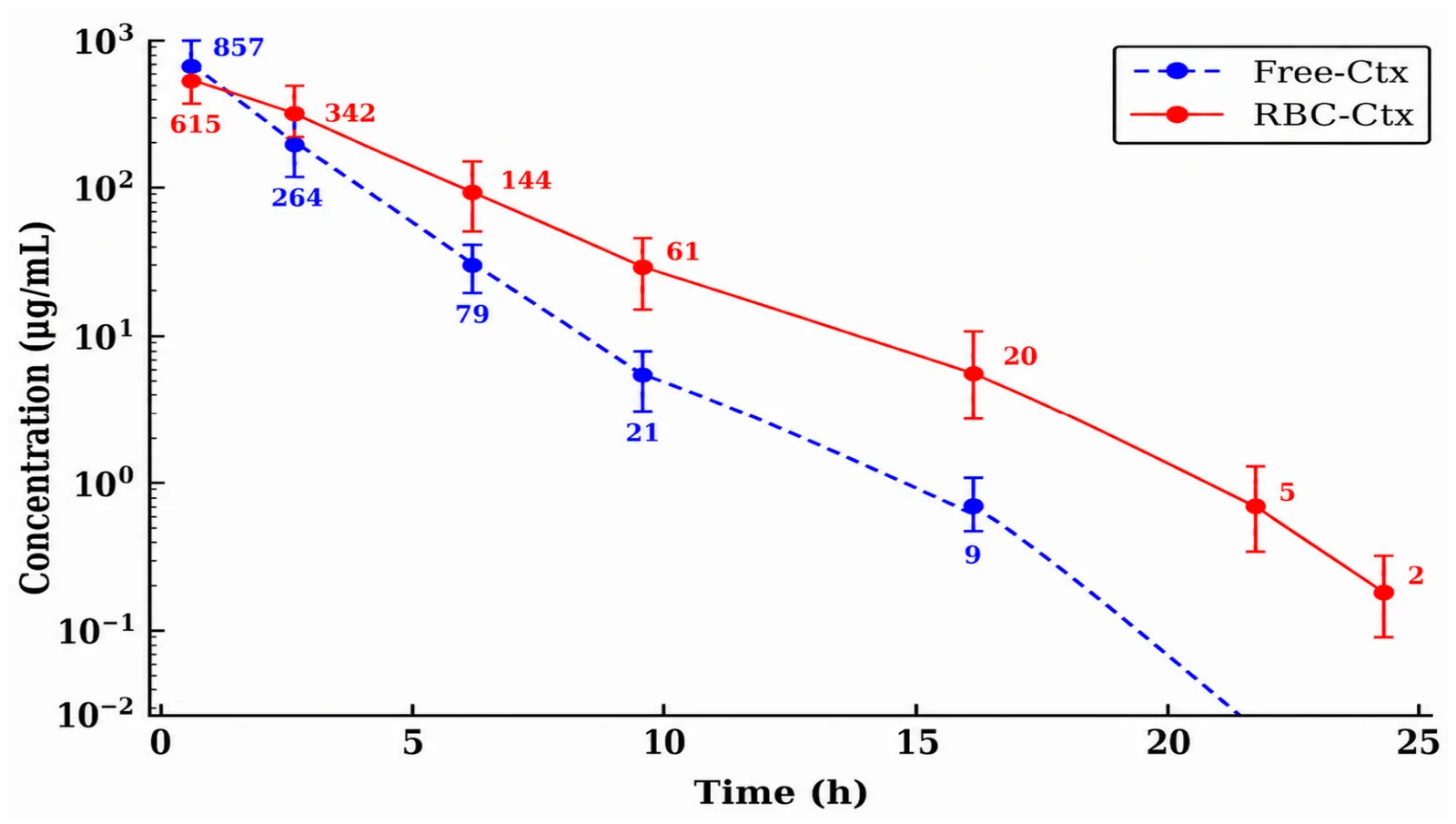
Plasma concentration levels of ceftriaxone in rats following intravenous administration of ceftriaxone encapsulated in erythrocyte carriers (RBC-Ctx) and free drug (Free-Ctx). Note: Concentrations are presented as mean ± standard error of the mean (SEM), *n* = 6 per group. Numerical concentration values are provided for each time point. The *Y*-axis is displayed on a semi-logarithmic scale.

Figure 5 presents concentration–time curves in semi-logarithmic coordinates for ceftriaxone administered to experimental animals in the Free-Ctx and RBC-Ctx groups.

In Table 2, the main pharmacokinetic constants of ceftriaxone encapsulated in erythrocyte carriers, as well as those of free ceftriaxone following intravenous administration, are presented.

The results of the population compartmental analysis were evaluated separately from the NCA results. Population parameter estimates for the RBC-Ctx two-compartment model and the corresponding model diagnostics were evaluated.

As shown by the obtained data, the use of RBC-Ctx results in fundamental alterations in the pharmacokinetics of ceftriaxone compared with the free drug. The elimination half-life (T½) increased approximately 2.5-fold, reaching 4.44 h, and, accordingly, the mean residence time (MRT_0_–∞) approximately doubled. The total exposure, expressed as the area under the concentration–time curve (AUC_0_–∞), increased by more than 30%. In parallel, total clearance (CL) decreased by nearly 30%, while the elimination rate constant (k_el) was reduced by approximately 2.4-fold. With intravenous administration of RBC-Ctx, compared with intravenous administration of free ceftriaxone (Free-Ctx), the apparent volume of distribution parameters of ceftriaxone in experimental animals (Vss and Vd) increased substantially, by 58% and 84%, respectively. For ceftriaxone encapsulated in erythrocytes (RBC-Ctx), the transfer rate constant (k_12_) from the central compartment (blood) to the peripheral compartment (tissues) and the reverse transfer rate constant (k_21_) from peripheral tissues back to the blood were determined. Comparison of these rate constants suggests that the drug may be retained in peripheral tissues, as k_21_ is substantially lower than k_12_.

Thus, under the experimental conditions, intravenous administration of RBC-Ctx loaded with ceftriaxone results in a pharmacokinetic profile characterized by prolonged drug release, as evidenced by increased T½, AUC_0_–∞, and MRT_0_–∞, along with decreased CL and k_el. Prolonged release of ceftriaxone into the plasma can be explained by the fact that ceftriaxone is a polar drug and, once internalized within erythrocytes, it does not readily diffuse across the cellular membrane due to its polar nature. Thus, drug release is expected to occur primarily through lysis of the erythrocyte carriers, as previously suggested for gentamicin [18]. The observed substantially higher value of the ceftriaxone transfer rate constant from the central compartment to the peripheral compartment compared with the reverse transfer constant, as well as the marked increase in the apparent volume of distribution of ceftriaxone (Vss and Vd), raises the question of potential significant differences in the biodistribution of ceftriaxone administered as RBC-Ctx compared with Free-Ctx. Accordingly, we analyzed the distribution of the studied formulations in selected peripheral tissues (Table 3).

Table 3 summarizes the tissue-to-plasma ratios calculated from the mean values obtained for the corresponding organs 1 h and 12 h after intravenous administration in the Free-Ctx and RBC-Ctx groups.

As shown in Table 3, the tissue-to-plasma concentration ratios (T/P ratios) at 1 h differed between the Free-Ctx and RBC-Ctx groups. The most pronounced differences between the two formulations were observed in the liver, spleen, and kidney, whereas the T/P ratios in the lung, heart, pancreas, and skeletal muscle were broadly comparable. These T/P ratios are intended only as relative indices of ceftriaxone distribution and should not be interpreted as validated absolute tissue concentrations. For calculating the tissue-to-plasma concentration ratio at 12 h, the plasma concentration was approximately estimated from the fitted concentration–time pharmacokinetic curve (Figure 5).

Table 4 summarizes the ratios of the mean values obtained at 1 after intravenous administration for the corresponding organs in the RBC-Ctx and Free-Ctx groups.

As shown in Table 4, relative within-organ comparison revealed differences between the RBC-Ctx and Free-Ctx groups. The RBC-Ctx/Free-Ctx ratio was below 1 in the kidney (0.68) and above 1 in the liver (2.58), spleen (5.61), lung (1.27), heart (1.31), pancreas (1.47), and skeletal muscle (1.38). The largest relative difference between the two formulations was observed in the spleen, followed by the liver. These ratios are intended only for within-organ comparison between RBC-Ctx and Free-Ctx and should not be interpreted as absolute tissue concentrations or as quantitative comparisons between different organs.

Relative tissue distribution of ceftriaxone 1 h after intravenous administration, expressed as the ratio of mean analytical values in the RBC-Ctx and Free-Ctx groups (RBC-Ctx/Free-Ctx) for each organ, is shown in Table 4.

Relative tissue distribution at 12 h was not presented because the mean Free-Ctx analytical values in several organs were zero or close to zero, which prevented reliable calculation of the ratio of RBC-Ctx/Free Ctx. Therefore, the relative tissue comparison was restricted to the 1 h time point.

Measurable concentrations of ceftriaxone at 12 h after administration in the Free-Ctx group were observed only in the kidneys (0.0065 ± 0.0007 mg/g). In contrast, measurable ceftriaxone concentrations were detected in multiple tissues in the RBC-Ctx group at 12 h after administration: Spleen (0.0382 ± 0.0028 mg/g); Liver (0.0124 ± 0.0014 mg/g); Lungs (0.0089 ± 0.0005 mg/g); Kidney (0.0055 ± 0.0007 mg/g). Thus, compared with free ceftriaxone, RBC-Ctx was associated with more sustained tissue detectability of ceftriaxone over the 12 h observation period, whereas concentrations in animals receiving free ceftriaxone declined markedly or became undetectable during the same period.

## 4. Discussion

Collectively, these findings indicate that erythrocyte-mediated delivery alters the relative distribution pattern of ceftriaxone compared with conventional free-drug administration. Furthermore, tissue-to-plasma concentration ratios (T/P ratios) of ceftriaxone in the RBC-Ctx group differed from those observed in the control group, with the most pronounced differences observed in the liver, spleen, and kidney. The observed differences in the relative distribution pattern are of particular interest given the immunological functions of these organs. The liver and spleen represent major components of the reticuloendothelial system (RES), harboring abundant populations of phagocytic cells responsible for the clearance of circulating pathogens and particulate materials. In addition, the lungs possess a highly specialized immune microenvironment in which both tissue-resident memory T cells and innate immune cells contribute to protection against respiratory infections and inflammatory diseases [38,39]. Consequently, these physiological characteristics may contribute to the differences in relative distribution observed between RBC-Ctx and Free-Ctx. Accordingly, the differences in tissue-to-plasma concentration ratios (T/P ratios) following administration of ceftriaxone-loaded erythrocyte carriers (RBC-Ctx), relative to the Free-Ctx group, may reflect altered disposition of the carrier erythrocytes within these organs. However, because matrix-specific bioanalytical validation was not performed for individual tissue matrices, these findings should be interpreted as relative distribution indices rather than validated absolute tissue concentrations.

The increased apparent volumes of distribution observed for RBC-Ctx should not be interpreted as a physical volume occupied by the carrier erythrocytes. Pharmacokinetic volumes of distribution are apparent parameters reflecting the relationship between the amount of drug in the body and its measured plasma concentration. The Vss value observed in the RBC-Ctx group (approximately 688 mL/kg) is close to the reported total body water volume in rats and exceeds both blood and extracellular fluid volumes, suggesting extensive redistribution of ceftriaxone beyond the vascular compartment. This interpretation is also compatible with the differences in relative tissue distribution observed at 1 h, as reflected by the tissue-to-plasma concentration ratios (T/P ratios).

Overall, our results indicate that incorporation of ceftriaxone into autologous erythrocytes substantially modifies its pharmacokinetic profile in rats and is associated with differences in relative tissue distribution. Compared with free ceftriaxone (Free-Ctx), administration of RBC-Ctx resulted in a prolonged plasma elimination half-life and a significant increase in systemic drug exposure, as reflected by the larger area under the plasma concentration–time curve (AUC). These findings suggest sustained drug release from the erythrocyte carriers and prolonged circulation of ceftriaxone in vivo. The prolonged systemic exposure of ceftriaxone encapsulated within autologous erythrocytes, together with the observed differences in tissue-to-plasma concentration ratios (T/P ratios) at 1 h, is consistent with altered in vivo disposition of ceftriaxone following erythrocyte-mediated delivery. Such a pharmacokinetic profile may facilitate preferential targeting of RES-associated tissues through phagocytic uptake of the loaded erythrocytes. The differences in relative distribution observed following RBC-Ctx administration suggest that erythrocyte-based carriers may offer an alternative to conventional drug delivery platforms, including nanoparticle-based systems. This strategy may be particularly beneficial for the treatment of intracellular infections caused by cephalosporin-sensitive pathogens, where efficient delivery of antibiotics to phagocyte-rich tissues is desirable. Similar biodistribution findings have previously been reported for the antibiotic amikacin encapsulated within autologous erythrocytes [19].

In the present study, we characterized the pharmacokinetics of erythrocyte-encapsulated ceftriaxone following intravenous administration to healthy rats, i.e., animals without evidence of inflammatory or infectious processes. We have reason to believe that the biodistribution profile of RBC-Ctx observed in healthy animals may differ substantially in the presence of an inflammatory or infectious focus. Changes in vascular permeability, activation of phagocytic cells, and alterations in reticuloendothelial system activity associated with inflammation may influence the tissue distribution and retention of erythrocyte-based carriers. Beyond the pharmacokinetic advantages described above, erythrocytes possess several unique features that make them attractive drug delivery vehicles. As naturally circulating cells, erythrocytes can access virtually all vascularized tissues in the body, enabling widespread distribution of associated therapeutics. At the same time, erythrocyte-based carriers may influence drug distribution toward tissues rich in reticuloendothelial system (RES) components owing to their recognition and uptake by resident phagocytic cells. This combination of systemic accessibility and potential tissue selectivity represents a distinct advantage over many conventional drug delivery platforms.

It should be noted that the present study has several limitations. The calibration procedure used for ceftriaxone quantification was solution-based, with calibration standards prepared in physiological saline, rather than matrix-matched using plasma or tissue homogenates. Therefore, matrix-specific effects on ceftriaxone recovery and quantification were not directly assessed. The absence of matrix-matched calibration and matrix-specific validation represents an important analytical limitation of the present study, particularly with respect to the quantitative interpretation of ceftriaxone concentrations in individual tissues. Nevertheless, previously validated analytical methods have reported high extraction recovery of ceftriaxone from plasma. Kim and Lee reported an overall ceftriaxone recovery of 90.9 ± 3.8% from rat plasma following protein precipitation with acetonitrile [40], while Hussein and Hammami reported ceftriaxone extraction recovery of ≥94% (mean 96%) from human plasma [41]. These published findings support the plausibility of the plasma pharmacokinetic trends observed in the present study; however, they do not substitute matrix-specific validation of the analytical method. Previous studies have demonstrated the feasibility of determining ceftriaxone in tissue samples using HPLC-based methods with solution-based calibration. These studies reported high extraction recoveries and acceptable analytical performance, providing a literature precedent for this analytical approach in the assessment of ceftriaxone tissue distribution. For example, Martin et al. reported substantial penetration of ceftriaxone into mediastinal and cardiac tissues following intravenous administration [42,43]. Martin et al. also investigated ceftriaxone concentrations in abdominal tissues during pancreatic surgery [44] and in abdominal tissues during open prostatectomy [45]. These studies demonstrate that ceftriaxone can be quantitatively determined in different tissue samples using HPLC-based analytical procedures with a reported recovery of 98 ± 5% and acceptable intra- and inter-day reproducibility. Similarly, other clinical studies have investigated ceftriaxone concentrations at surgical sites and in bone tissue, including studies by Kundra et al. [46], Gergs et al. [47], and Garazzino et al. [48]. A reported recovery for bone samples was 86%. The study by Sheikh et al. also demonstrated substantial ceftriaxone recovery and measurable concentrations in serum and tissue samples obtained from pediatric patients undergoing surgery [49]. The study used ceftriaxone standard-solution calibration and reported QC accuracy of 94–104% and intra- and inter-day coefficients of variation of 1–6%. In addition, Li et al. described an analytical approach for determination of free ceftriaxone concentration and its application to prediction of lung tissue concentration [50].

These studies demonstrate that non-tissue-specific calibration approaches have been employed for ceftriaxone analysis in tissue samples, provided that appropriate analytical performance characteristics have been established. Nevertheless, we acknowledge that, in the absence of tissue-specific matrix-matched calibration and comprehensive assessment of recovery and matrix effects, the absolute tissue concentrations should be interpreted with caution and should not be regarded as fully validated quantitative values.

Although representative chromatograms of blank plasma and tissue samples showed no apparent interfering peaks at the ceftriaxone retention time, a comprehensive assessment of matrix effects was not performed. The available calibration characteristics and analytical data are provided in the Supplementary Information. Therefore, the quantitative plasma and tissue concentration data should be interpreted with consideration of these methodological limitations.

Further studies using matrix-matched calibration and comprehensive bioanalytical validation are warranted to confirm the accuracy, precision, recovery, and matrix effects associated with ceftriaxone quantification in plasma and individual tissue matrices.

## 5. Conclusions

In conclusion, encapsulation of ceftriaxone within erythrocyte carriers significantly modified its pharmacokinetic profile in rats, resulting in prolonged circulation and increased systemic exposure, and was associated with differences in relative tissue distribution compared with free ceftriaxone. These results suggest that erythrocyte-based drug delivery systems may provide an effective approach for extending the duration of antibiotic activity and enhancing drug delivery to phagocytic cell populations, thereby offering potential advantages for the treatment of infections involving intracellular or RES-associated pathogens. Novel strategies are emerging that exploit the unique structural and functional properties of erythrocytes, including intact red blood cells, erythrocyte ghosts (red blood cell membranes), and hemoglobin itself [16]. Additional approaches involve the genetic engineering and surface modification of erythrocytes [51,52], modulation of the immune system using erythrocyte-associated antigens [53,54], and vascular delivery of erythrocyte-bound nanocarriers via the erythrocyte hitchhiking strategy [24,55].

In this study, we utilized an optimized and simplified erythrocyte drug-loading approach that is compatible with transfusion-grade blood products and is readily amenable to automation and large-scale manufacturing. Although several innovative erythrocyte-based delivery strategies have recently emerged, our pharmacokinetic data indicate that the established erythrocyte-mediated targeted drug delivery system (TDDS) retains considerable translational potential and may facilitate clinical implementation aimed at improving the efficacy of antibiotic therapy through optimized pharmacokinetic behavior and targeted drug delivery.

Nevertheless, we acknowledge that, as with virtually all antibiotic-targeted drug delivery systems (TDDSs), erythrocyte-based carriers are subject to several limitations, including premature clearance from the circulation, inactivation before reaching the target site, limited targeting accuracy, and lower therapeutic efficacy in inflammatory conditions than would be anticipated from pharmacokinetic profiles determined under normal physiological conditions. Enhancing the precision and efficiency of antibiotic delivery therefore remains a major challenge in pharmacotherapy. Further studies using experimental models of infection and inflammation are required to optimize erythrocyte-based antibiotic delivery systems and to better define their therapeutic potential. Encapsulation of ceftriaxone within autologous erythrocytes altered its release kinetics, pharmacokinetic profile, and tissue distribution. Erythrocyte-mediated delivery reduced the rate of drug elimination, prolonged systemic circulation, and modified tissue bioavailability compared with free ceftriaxone. Owing to the physiological function of the reticuloendothelial system (RES), erythrocyte-mediated delivery was associated with differences in the relative tissue distribution of ceftriaxone, particularly in the liver and spleen. Experimental pharmacokinetic analysis demonstrated that erythrocyte encapsulation significantly increased the elimination half-life (T½), area under the plasma concentration-time curve (AUC), and mean residence time (MRT), while reducing total body clearance (CL) and altering the apparent volume of distribution relative to the free drug. These findings indicate prolonged systemic exposure and sustained circulation of ceftriaxone following erythrocyte-mediated delivery. Tissue distribution studies further indicated differences in the relative distribution pattern of ceftriaxone between the RBC-Ctx and Free-Ctx groups, particularly in RES-rich organs such as the liver and spleen. Measurable ceftriaxone concentrations were detected in these tissues at 12 h after a single intravenous dose, whereas ceftriaxone concentrations in animals receiving the free drug declined markedly or became undetectable during the same period.

Collectively, these findings demonstrate that encapsulation of ceftriaxone into autologous erythrocytes effectively modulates its pharmacokinetic behavior by prolonging systemic exposure, reducing drug clearance, and altering the relative tissue distribution of ceftriaxone. This erythrocyte-based delivery strategy therefore represents a promising approach for targeted antibiotic delivery and pharmacokinetic optimization, particularly for infections involving RES-associated organs.

## Figures and Tables

**Table 1:** Analytical performance characteristics of the HPLC–UV method for ceftriaxone determination.

Parameter	Value
Linear dynamic range (mg/L)	0.5–50
Regression equation (Y)	Y = a + bC, where C is ceftriaxone concentration (mg/L)
Slope (b)	0.3521
Intercept (a)	0.0130
Correlation coefficient (r)	0.9999
Limit of detection (LOD, mg/L)	0.3925
Limit of quantification (LOQ, mg/L)	1.1895
Relative standard deviation (RSD, %)	0.7975

**Table 2:** Pharmacokinetic parameters of ceftriaxone in rats following a single intravenous administration in the Free-Ctx and RBC-Ctx groups at a dose of 340 mg/kg.

Parameter	Free-Ctx (Mean ± SD)	RBC-Ctx (Mean ± SD)
AUC_0_–∞ (mg·h/mL)	1.18 ± 0.18	1.56 ± 0.081 ***
AUC_0_–t_l_ₐ_st_ (mg·h/mL)	1.16 ± 0.18	1.55 ± 0.081 ***
C_0_ (mg/mL)	1.07 ± 0.13	0.70 ± 0.13 ***
CL (mL/h/kg)	294.0 ± 48.8	218.2 ± 11.0 **
C_last (mg/mL)	0.009 ± 0.0028	0.002 ± 0.0004 ***
C_max (mg/mL)	0.84 ± 0.064	0.62 ± 0.085 ***
T½ (h)	1.79 ± 0.14	4.44 ± 0.57 ****
k_el (h^−1^)	0.39 ± 0.03	0.16 ± 0.021 ****
MRT_0_–∞ (h)	1.51 ± 0.22	3.15 ± 0.21 ****
V_ss (mL/kg)	435.0 ± 23.1	688.3 ± 61.0 ****
V_d (mL/kg)	755 ± 118	1397 ± 183 ****
k_12_ (h^−1^)	—	2.97
k_21_ (h^−1^)	—	0.36

Note: Statistical significance between groups was assessed using an unpaired two-tailed *t*-test. ** *p* < 0.01; *** *p* < 0.001; **** *p* < 0.0001. AUC_0_–∞—area under the concentration–time curve extrapolated to infinity; AUC_0_–t_l_ₐ_st_—area under the concentration–time curve up to the last measurable point; C_0_—initial concentration; CL—clearance; C_last—last measured concentration; C_max—maximum concentration; T½—elimination half-life; k_el—elimination rate constant; MRT_0_–∞—mean residence time; V_ss—volume of distribution at steady state; V_d—volume of distribution; k_12_ и k_21_—micro-rate constants for intercompartmental transfer.

**Table 3:** Tissue-to-plasma concentration ratios of ceftriaxone 1 h and 12 h after intravenous administration in the Free-Ctx and RBC-Ctx groups.

Organ	Tissue-to-Plasma Ratios 1 h After Administration		Tissue-to-Plasma Ratios 12 h After Administration	
	Free-Ctx T/P	RBC-Ctx T/P	Free-Ctx T/P	RBC-Ctx T/P
Kidney	4.96	2.6	0.43	0.14
Liver	0.57	1.13	0	0.31
Spleen	0.17	0.75	0	0.95
Lung	0.27	0.26	0	0.22
Heart	0.31	0.32	0	0
Pancreas	0.14	0.16	0	0
Muscle	0.16	0.17	0	0

Note. T/P ratios were calculated as the ratio of the mean tissue value to the corresponding mean plasma value at 1 h and 12 h. To calculate the tissue-to-plasma ratios at 12 h, the predicted ceftriaxone plasma con-centration was determined from the mean concentration–time curve shown in Figure 5. The values are presented as relative distribution indices and should not be interpreted as absolute tissue concentrations.

**Table 4:** Relative tissue distribution of ceftriaxone at 1 after intravenous administration, expressed as the ratio of mean values between the RBC-Ctx and Free-Ctx groups.

Organ	RBC-Ctx/Free-Ctx Ratio
Kidney	0.68
Liver	2.58
Spleen	5.61
Lung	1.27
Heart	1.31
Pancreas	1.47
Muscle	1.38

Note. Ratios were calculated from the mean analytical responses obtained for each corresponding organ in the RBC-Ctx and Free-Ctx groups. The ratios are dimensionless and are intended only for relative within-organ comparison between the two formulations; they should not be interpreted as absolute tissue concentrations.

## Data Availability

The original contributions presented in this study are included in the article. Further inquiries can be directed to the corresponding authors.

## Supplementary Materials

The following supporting information can be downloaded at https://www.mdpi.com/article/10.3390/pharmaceutics18091052/s1, Figure S1. Representative chromatograms of blank plasma obtained from untreated animals; Figure S2. Representative chromatograms of blank tissue samples (liver) obtained from untreated animals; Figure S3. Representative HPLC-UV chromatogram of a ceftriaxone standard solution and the corresponding solution-based external calibration curve. Ceftriaxone was detected at a retention time of 8.073 min at 260 nm. The calibration curve was linear over the investigated concentration range (0.5–50 mg/L), with a slope of 0.3525 and a coefficient of determination (R^2^) of 0.99995.

## Abbreviations

The following abbreviations are used in this manuscript:

Ctx	Ceftriaxone
TDDS	Targeted drug delivery systems
RBC-Ctx	Ceftriaxone encapsulated in autologous erythrocytes
Free-Ctx	Free ceftriaxone
RBC	Red blood cell
IL-1β	Interleukin-1β
IACUC	Institutional Animal Care and Use Committee
r	Correlation coefficient
LOD	Limit of detection
LOQ	Limit of quantification
RSD	Relative standard deviation
HPLC	High-performance liquid chromatography
HPLC-UV	High-Performance Liquid Chromatography-Ultraviolet
k12 and k21	Intercompartmental transfer rate constants
Vss	Steady-state volume of distribution
Vd	Apparent volume of distribution
T/P ratio	Tissue-to-plasma concentration ratio
PBS	Phosphate-buffered saline
mean ± SD	Mean ± standard deviation
ESR	Erythrocyte sedimentation rate
AUC_0_–∞	Area under the concentration–time curve extrapolated to infinity
AUC_0_–t_l_ₐ_st_	Area under the concentration–time curve up to the last measurable point
C_0_	Initial concentration
CL	Clearance
C_last	Last measured concentration
C_max	Maximum concentration
T½	Elimination half-life
k_el	Elimination rate constant
MRT_0_–∞	Mean residence time
