# Supplementary material for: Pharmacokinetics of Ceftriaxone Encapsulated in Carrier Erythrocytes in Experimental Study
Source: Pharmaceutics. 2026 Aug 25;18(9):1052. doi: 10.3390/pharmaceutics18091052 (PMC13610815; doi:10.3390/pharmaceutics18091052)
Supplement: Supplementary file 1 [file pharmaceutics-18-01052-s001.zip › pharmaceutics-4447246-supplementary.pdf]

Representative chromatograms of plasma and tissue samples (liver) obtained from animals.

Chromatogram and Results

Injection Details

Injection Name:	**5,12**	*Run Time (min):*	**9,00**
Vial Number:	**RC3**	*Injection Volume:*	**2,00**
Injection Type:	**Unknown**	*Channel:*	**UV_VIS_1**
Calibration Level:		*Wavelength:*	**260**
Instrument Method:	**Ceftriaxone4**	*Bandwidth:*	**1**
Processing Method:	**Quantitative**	*Dilution Factor:*	**1,0000**
Injection Date/Time:	**07.окт.24 17:41**	*Sample Weight:*	**1,0000**

Chromatogram

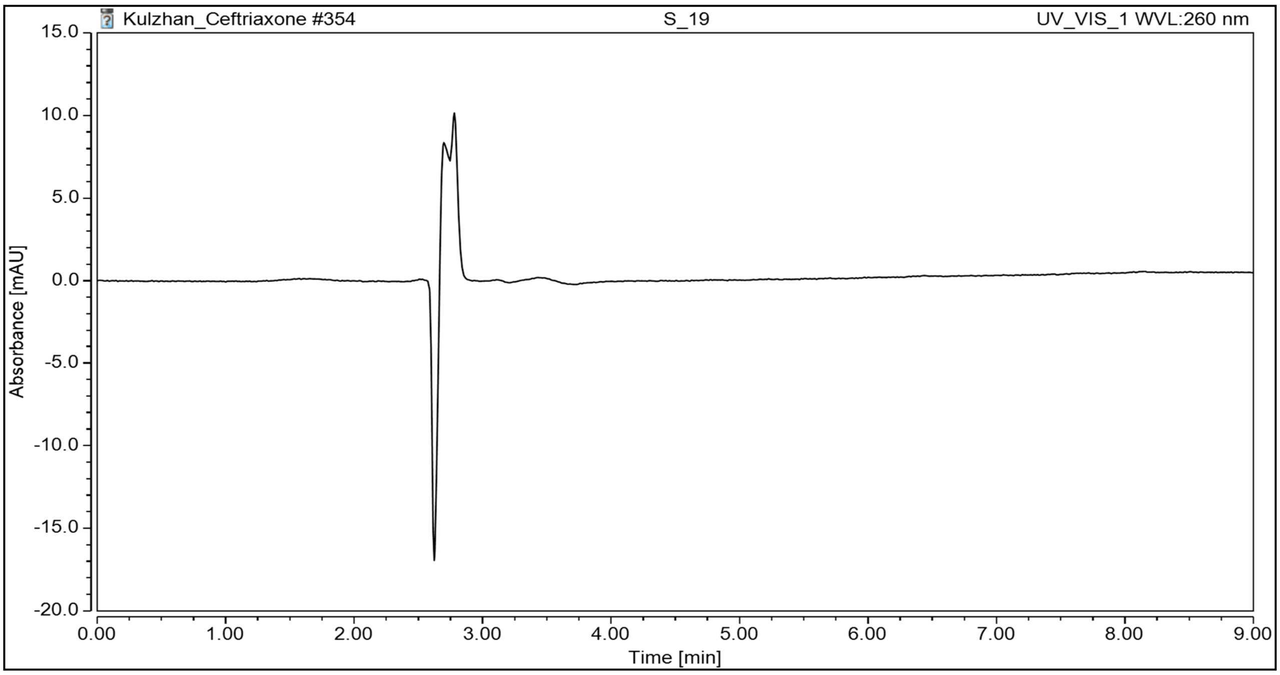

Integration Results

No.	Peak Name	Retention Time min	Area mAU*min	Height mAU	Relative Area %	Relative Height %	Amount ppm
n.a.	Ceftriaxone	n.a.	n.a.	n.a.	n.a.	n.a.	n.a.
**Total:**			**0,000**	**0,000**	**0,00**	**0,00**	

Figure S1. Representative chromatograms of blank plasma obtained from untreated animals

Chromatogram and Results

Injection Details

Injection Name:	**5,12**	*Run Time (min):*	**12,00**
Vial Number:	**RA7**	*Injection Volume:*	**2,00**
Injection Type:	**Unknown**	*Channel:*	**UV_VIS_1**
Calibration Level:		*Wavelength:*	**260**
Instrument Method:	**Ceftriaxone4**	*Bandwidth:*	**1**
Processing Method:	**Quantitative**	*Dilution Factor:*	**1,0000**
Injection Date/Time:	**28.окт.24 13:37**	*Sample Weight:*	**1,0000**

Chromatogram

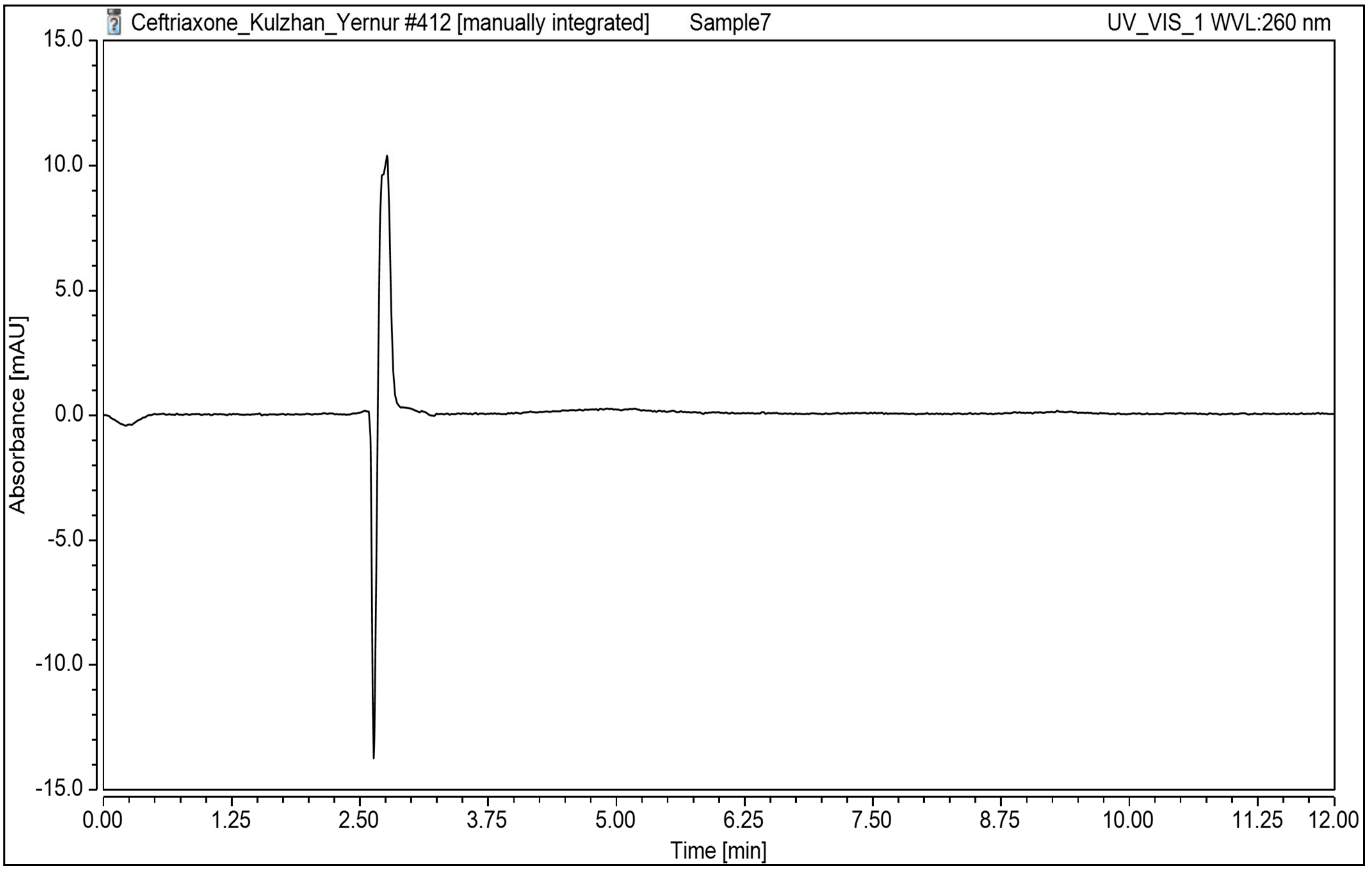

Integration Results

No.	Peak Name	Retention Time min	Area mAU*min	Height mAU	Relative Area %	Relative Height %	Amount ppm
n.a.	Ceftriaxone	n.a.	n.a.	n.a.	n.a.	n.a.	n.a.
**Total:**			**0,000**	**0,000**	**0,00**	**0,00**	

Figure S2. Representative chromatograms of blank tissue samples (liver) obtained from untreated animals

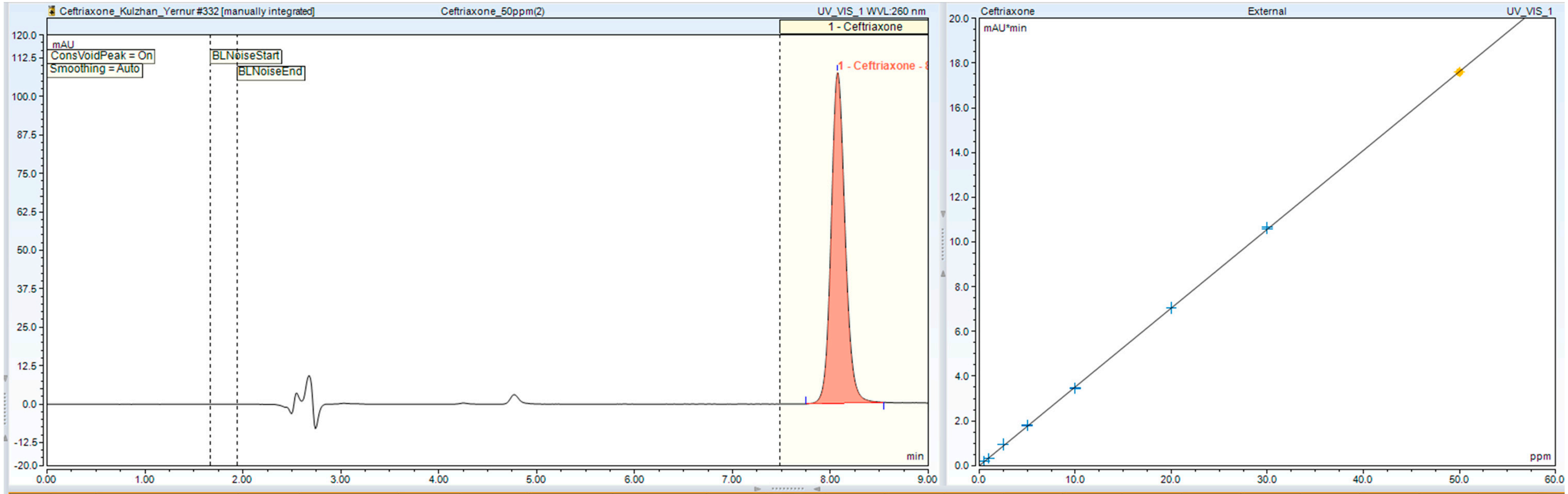

Peak No.	Peak Name	Ret.Time min	Cal.Type	Eval.Type	Number of Points	Rel.Std.Dev. %	Coeff.of Determination	C0 (Offset)	C1 (Slope)	C2 (Curve)
1	Ceftriaxone	8.073	Lin	Area	16	0.7975	0.99995	0.0000	0.3525	0.0000
Maximum						0.7975	0.99995			
Minimum						0.7975	0.99995			

Figures S3. Representative HPLC-UV chromatogram of a ceftriaxone standard solution and the corresponding solution-based external calibration curve. Ceftriaxone was detected at a retention time of 8.073 min at 260 nm. The calibration curve was linear over the investigated concentration range (0.5–50 mg/L), with a slope of 0.3525 and a coefficient of determination (R^2) of 0.99995.
